# Charting a killer course to the solid tumor: strategies to recruit and activate NK cells in the tumor microenvironment

**DOI:** 10.3389/fimmu.2023.1286750

**Published:** 2023-11-08

**Authors:** Ana L. Portillo, Jonathan K. Monteiro, Eduardo A. Rojas, Tyrah M. Ritchie, Amy Gillgrass, Ali A. Ashkar

**Affiliations:** ^1^ Department of Medicine, McMaster University, Hamilton, ON, Canada; ^2^ McMaster Immunology Research Centre, McMaster University, Hamilton, ON, Canada; ^3^ Centre for Discovery in Cancer Research, McMaster University, Hamilton, ON, Canada

**Keywords:** natural killer cells, cancer immunotherapy, solid tumors, tumor microenvironment, oncolytic virus, nanomedicine

## Abstract

The ability to expand and activate natural Killer (NK) cells *ex vivo* has dramatically changed the landscape in the development of novel adoptive cell therapies for treating cancer over the last decade. NK cells have become a key player for cancer immunotherapy due to their innate ability to kill malignant cells while not harming healthy cells, allowing their potential use as an “off-the-shelf” product. Furthermore, recent advancements in NK cell genetic engineering methods have enabled the efficient generation of chimeric antigen receptor (CAR)-expressing NK cells that can exert both CAR-dependent and antigen-independent killing. Clinically, CAR-NK cells have shown promising efficacy and safety for treating CD19-expressing hematologic malignancies. While the number of pre-clinical studies using CAR-NK cells continues to expand, it is evident that solid tumors pose a unique challenge to NK cell-based adoptive cell therapies. Major barriers for efficacy include low NK cell trafficking and infiltration into solid tumor sites, low persistence, and immunosuppression by the harsh solid tumor microenvironment (TME). In this review we discuss the barriers posed by the solid tumor that prevent immune cell trafficking and NK cell effector functions. We then discuss promising strategies to enhance NK cell infiltration into solid tumor sites and activation within the TME. This includes NK cell-intrinsic and -extrinsic mechanisms such as NK cell engineering to resist TME-mediated inhibition and use of tumor-targeted agents such as oncolytic viruses expressing chemoattracting and activating payloads. We then discuss opportunities and challenges for using combination therapies to extend NK cell therapies for the treatment of solid tumors.

## 1 Introduction

Natural killer (NK) cells are a subset of innate lymphocytes that can directly kill target cells without prior antigen sensitization. NK cells were first recognized for this unique ability over five decades ago and have since been characterized as an integral part of the body’s innate immune defense against infection and cancer. NK cells are of particular interest as they can recognize and kill tumor cells while leaving healthy cells unharmed through recognition of HLA-class I molecules expressed on healthy cells (1). NK cell-based cancer immunotherapies leverage this unique ability and provide a safer alternative to other adoptive cell therapies like chimeric antigen receptor (CAR)-T cells which become activated against any cell expressing the target antigen. Indeed, various clinical trials have shown that *ex vivo* activated and expanded NK cells can achieve high clinical efficacy against hematologic malignancies with a lower risk for adverse effects such as graft vs host disease (2–4). Additionally, NK cells engineered to express a CAR have shown clinical efficacy against B-cell malignancies with limited therapy-induced toxicities such as cytokine release syndrome (5). CAR-NK cell therapies can also overcome tumor antigen escape problems seen with CAR-T cells due to complete loss or downregulation of antigen expression by target cells since CAR-NK cells can target tumors through CAR-independent mechanisms. These characteristics enable NK cells to be used as an effective allogenic “of-the-shelf” product reducing cost and time to treatment in comparison to CAR-T cell therapy. However, despite over a decade of pre-clinical and clinical development, the use of NK cell therapy against solid tumors remains limited. Solid tumors pose unique barriers to adoptive cell therapies including NK cells, by impairing immune cell entry into the tumor core and dampening anti-tumor effector functions within the immunosuppressive and metabolically hostile tumor microenvironment (TME). However, a more challenging hurdle is the tumor’s ability to block NK cell trafficking to the tumor core, leaving the highly efficient killers on the sidelines. In this review, we focus on summarizing the mechanisms blocking NK cell homing and infiltration to the solid tumor core and discuss various strategies that try to overcome these barriers and give access to NK cell adoptive cell therapy. This includes therapies that directly modulate the structural barriers by the tumor and that improve blood flow into the tumor. We also discuss strategies that modulate the chemokine-chemokine receptor axis to enhance the homing ability of NK cells to the tumor site. Lastly, we highlight how engineered oncolytic viruses (OVs) and nanotechnologies can offer unique advantages to other therapeutic modalities through their enhanced ability express or deliver immunomodulatory agents within the TME. OVs are of particular interest for synergizing with NK cells therapy as they offer a platform for expressing a wide range of chemoattracting and activation factors in the TME, while also contributing to tumor clearance through tumor debulking. The development of tumor selective therapies that are permissive to the tumor core and that can be modified to deliver NK cell recruitment and activation payloads will unleash the potential of NK cell adoptive therapies for solid tumors.

## 2 Natural Killer (NK) cell adoptive cell therapy

### 2.1 Ex vivo activation and genetic manipulation of NK cells for cancer immunotherapy

NK cells account for 10-15% of peripheral blood lymphocytes and play a key role in the first-line immune response against viral infection and malignancy (1). NK cells express a variety of different germ-line encoded activation receptors that recognize stress-induced ligands upregulated on the surface of target cells (6). Moreover, NK cells also express various inhibitory receptors such as the killer immunoglobulin-like receptors (KIRs) and NKG2A which prevent their activation towards normal cells overexpressing HLA-class I and HLA-E molecules (7). NK cell activation and their resultant effector function is orchestrated by a balance between activation and inhibitory signaling that occurs upon NK cell receptor engagement (7). NKG2D and Natural Cytotoxicity Receptors (NCRs; NKp30, NKp44, and NKp46) are major NK cell activation receptors for the detection and elimination of viral and malignant cells (7). NKG2D recognizes the ligands MIC-A, MIC-B, ULBP 1-6 proteins which are absent from the surface of healthy cells but are upregulated by infected or stressed cells (8). In addition to upregulation of stress ligands, virally infected cells and tumors can also downregulate HLA class I which render them sensitive to NK cell-mediated killing and untargeted by effector T cells. NK cells also mediate antibody-dependent cellular cytotoxicity (ADCC) by engagement of FcγRIII (CD16) (6). Upon activation, NK cells kill targets directly through the release of lytic granules including granzymes and perforin (6). NK cell activation also leads to the secretion of pro-inflammatory cytokines including interferon-γ (IFN-γ) and tumor necrosis factor-α (TNF-α) which further shape innate and adaptive immune responses (6). NK cell can also induce tumor killing independent of perforin and granzyme by engagement of Fas Ligand (FasL) and Tumor Necrosis Factor-Related Apoptosis Inducing Ligand (TRAIL) on NK cells with cognate receptors, Fas or TRAIL, expressed on target cells (7). Human NK cells are generally defined as CD3-CD56+, and traditionally, the extent of CD56 expression has been used to classify NK cells into two distinct functional subsets. The two subsets can be defined as the regulatory and high cytokine-producing CD56^bright^CD16- subset and the cytotoxic CD56^dim^CD16+ subset. The CD56^dim^ NK cell population is mature and accounts for 80-95% of NK cells in peripheral blood (9).

Developments in the *ex vivo* activation, expansion, and genetic engineering of NK cells over the last decade has enabled their use as a potent cancer immunotherapy. Different strategies have been developed for the clinical grade expansion of highly pure and activated NK cells including the use of cytokines (IL-2, IL-12, IL-15, and IL-21), or irradiated feeder cells such as K562 cells and plasma membrane particles expressing membrane bound (mb)-IL-21 or -IL-15 for co-stimulation (10–12). Small molecules for NK cell expansion and activation have also been investigated including the use of nicotinamide and a glycogen synthase kinase 3 inhibitor (13). NK cells from different sources including healthy donor or cancer patient peripheral blood, umbilical cord blood, NK cell lines and induced pluripotent stem cells (iPSC)-derived NK cells have been used to develop adoptive cell therapies (13). Developments in genetic engineering methods has allowed the efficient generation of scalable, clinical-grade NK cells products with superior anti-tumor functions. Various strategies are being pursued to increase NK cell cytotoxicity via gene manipulation including expression of exogenous cytokines, downregulation of inhibitory receptors, and expression of chimeric antigen receptors (CARs) or T cell receptors. Novel genetic engineering methods using clustered regularly interspaced short palindromic repeats (CRISPR)-associated protein (Cas9) gene editing and transduction with viral vectors such as adeno-associated virus (AAV) have enabled simultaneous insertion of CAR transgenes with long-term expression and gene knockouts in primary NK cells (14, 15). This is beneficial as CAR expression is target to a specific site on the genome compared to viral-based engineering methods such as lentivirus or retroviruses in which the integration site is uncontrolled. Other methods that do not require CRISPR engineering have been recently described such as the use of an AAV carrying a *Sleeping Beauty* transposon and electroporation of an mRNA encoding a transposase for permanent transgene integration (16). This method potentially offers a higher safety profile than other viral-based methods such as retroviruses (17). CAR-NK cell therapies in particular hold immense promise in targeting heterogenous solid tumors due to their ability to exert both antigen-independent and CAR-directed killing while remaining safe against healthy cells (5, 18). The use of CARs to specifically redirect NK cells towards tumor targets has already demonstrated high clinical efficacy against CD19-positive hematological malignancies without the severe toxicities and graft-vs-host disease seen with CAR-T cell therapies (5). Due to a higher safety profile, NK cell-based immunotherapies have an unparalleled potential for an “off-the-shelf” product reducing the cost and time of treatment administration. Further, NK cells maybe overcome tumor escape problems due to loss of the CAR-specific antigen, as seen after CAR-T cell therapy, as NK cells maintain antigen-independent activity. While relapse due to CAR-NK cell self-recognition after trogocytosis of the tumor antigen is possible, co-expression of a traditional activating CAR and a CAR with the signal domain from an inhibitory KIR can limit NK cell self-targeting enhancing treatment efficacy (19).

Clinical trials using *ex vivo* expanded NK cells have achieved therapeutic efficacies against multiple hematological malignancies such as high-risk myeloid cancers (2–4). However, there are more limited studies using NK cell-based adoptive cell therapies against solid tumors. Although the frequency of NK cells within solid tumor sites is typically lower compared to other immune cells such as cytotoxic CD8+ T cells, they exert potent anti-tumor functions (20). Indeed, a recent meta-analysis and systematic review by Nersesian et al. demonstrates that infiltration of NK cells is a positive prognostic factor in solid tumors and is associated with improved overall survival (21). Importantly, a lower risk of death was highly related to the location of NK cells within the tumor, with a lower effect seen in tumors where NK cells primarily localized around the tumor margin or in the stromal compartment as opposed to the intra-tumoral epithelial region (21). These studies exemplify the importance of NK cell anti-tumor immunity not only in hematologic malignancies but also solid tumors. In various pre-clinical studies, K562 mb-IL21 expanded NK cells have shown to be highly efficient at preventing tumor establishment in human ovarian, lung, and breast cancer models (22–24). However, these activated NK cells are limited at eliminating already established primary tumors that are not easily accessible such as breast cancer. The real-time biodistribution of fluorescently labeled NK cells expanded with K562 feeder cells has been investigated after intravenous administration to non-tumor bearing and human hepatocellular carcinoma (HCC) bearing mice (25). Transferred NK cells are detected primarily within the lungs within the first 4 hours of injection which decreased after 24 hours, after which NK cells were found to accumulate within the liver and persisted for 14-21 days (25). Near-infrared real-time tracking has been used to examine the biodistribution of adoptively transferred *ex vivo* expanded NK cells in human MDA-MB-231 triple negative breast cancer (TNBC) xenograft models (26). Using this technique Thanh Uong et al. found that expanded NK cells mainly trafficked to the lungs and not the primary tumor site, reaching the lungs within 30 minutes of intravenous administration (26). While NK cells were detected in the tumor by 4h, immunohistochemistry showed that NK cells primarily localized around the tumor margin as opposed to the tumor core (26). Thus, combining adoptive transfer of NK cells with additional strategies is needed to enhance their therapeutic response.

### 2.2 Combination of NK cell therapy with other anti-cancer strategies

NK cell anti-tumor functions can be harnessed to improve efficacy of conventional anti-cancer treatments such as radiotherapy and chemotherapy, as well as checkpoint blockade and targeted antibody therapies. Checkpoint blockade involves blocking the cytotoxic T lymphocyte antigen-4 or programmed death-1/programmed death ligand 1 (PD-1/PD-L1) pathways to promote T cell anti-tumor responses (27, 28). NK cells also contribute to higher anti-tumor responses during PD-1/PD-L1 blockade (29). Pembrolizumab, a monoclonal antibody binding PD-1, is a first-line treatment for PD-L1 expressing non-small cell lung cancer (NSCLC). A phase II clinical trial has shown that combining NK cells and pembrolizumab significantly improved the overall survival of patients with NSCLC compared to checkpoint inhibitor monotherapy (30). Co-administration of monoclonal antibodies (mAbs) targeting tumor associated antigens (TAAs) and NK cells has also garnered clinical interest as a way to engage NK cell-mediated ADCC. The most common TAAs targeted in clinical trials for solid tumors are GD2, epidermal growth factor receptor (EGFR), and human epidermal growth factor receptor-2 (HER-2) (13). Completed clinical trials using trastuzumab (anti-HER2 mAb) or cetuximab (anti-EGFR mAb) in combination with NK cell therapy show these therapies are well tolerated (31, 32). In a phase I/II clinical trial, NK cell administration with cetuximab also significantly improved overall survival in patients with NSCLC (31).

NK cells can also be combined with traditional anti-cancer therapies. Patin et al. has shown that radiotherapy and inhibiting the DNA damage response pathway can synergize to enhance NK cell activity and tumor infiltration in a mouse model of head and neck squamous cell carcinoma (HNSCC) (33). Another recent study demonstrated that radiotherapy enhances the anti-tumor efficacy of a fused IL-2 and anti-PD-1 cytokine in HNSCC tumors (34). In this model, combination therapy enhanced NK cell activity leading to reduced tumor metastasis in the lungs. Another study showed that local irradiation of the MDA-MB-231 tumors can improve human expanded NK cell infiltration into the tumors. Combination therapy with radiation and NK cells led to significantly higher suppression of primary and metastatic tumor growth compared to the NK or irradiation only groups (35). Further, CD56-positive NK cells were only detected within primary tumor tissue in the group that received irradiation therapy prior to NK cell administration (35). While radiotherapy can enhance expression of NK cell activation ligands, it can also make tumor cells resistant to perforin-mediated apoptosis reducing their sensitivity to NK cell targeting 72h post-irradiation (36). Further, radiation can increase expression of PD-L1 and reduce NKG2D ligands on tumor cells inhibiting NK cell killing which is more pronounced with increased radiation dosing (37). Hence, determining optimal dosing and timing of radiotherapy regimens will be important when combining them with NK cell therapy. Pre-treatment with chemotherapies has also shown to sensitize tumor cells to NK cell-mediated killing. In a clinical study assessing adoptive transfer of NK cells with chemotherapy combination therapy, NK cell administration after chemotherapy significantly increased progression-free survival of patients with stage III advanced colon cancer (38). Overall, there are currently many ongoing clinical trials assessing non-engineered and engineered NK cells alone or in combination with other therapies for solid tumors and have been reviewed elsewhere (13, 39).

## 3 Barriers limiting NK cell trafficking, infiltration, and activation in the solid tumor

Despite the promising potential for combination of NK cells and already clinically approved anti-cancer therapies or other modalities, their efficient infiltration to the tumor core remains a challenge. There are various major factors that impair NK cell trafficking and infiltration into the solid tumor including the extensive extracellular matrix (ECM) barrier surrounding the tumor bed, the aberrant tumor vasculature leading to poor tumor perfusion, and a mismatch in the chemokine expression pattern within the TME to attract activated NK cells to the tumor. In this section we will summarize how these mechanisms work together to prevent efficient NK cell entry into the tumor bed and how the immunosuppressive TME impacts NK cell function inside the tumor. Enhancing NK cell infiltration into the tumor core and maintaining their anti-tumor function will be crucial to achieve durable responses with NK cell therapies for solid tumors.

### 3.1 The tumor stroma creates a rigid barrier around the tumor core

The solid tumor is a complex tissue made up tumor cells, a variety of immunosuppressive immune cells and the tumor stroma which consists of the abundant ECM, basement membrane, CAFs and endothelial cells. To enter the solid tumor site, immune cells first migrate towards the tumor through a cytokine/chemokine gradient. However, once they reach the tumor, they are shielded from the actual tumor core by the rigid and stiff ECM barrier (40). Thus, immune cells must migrate around the barrier and past ECM-adhesion sites to reach the tumor cells (40). Within the stroma, immune cells are unable to make direct contact with tumor cells and exert their anti-tumor functions. Tumor cells can themselves produce extracellular matrix components such as collagenases and ECM-modifying enzymes (41). Tumor cells also induce the differentiation of stromal cells into CAFs through secretion of the different growth factors including transforming growth factor β (TGF-β) and basic fibroblast growth factor (bFGF) (40). CAFs are abundant in solid tumors, making up to 80% of the tumor mass in some cancers (42). Within the tumor, CAFs are main contributors to ECM deposition, can produce TGF-β to support tumor growth and drive epithelial to mesenchymal transition leading to tumor invasiveness and metastasis (40). CAFs can be defined using a combination of morphological characteristics, genetic and protein biomarkers (43). For example, common biomarkers expressed by CAFs include alpha-smooth muscle actin (α-SMA), fibroblast activation protein (FAP), and platelet-derived growth factor alpha (43). However, these markers are not specific as they are also expressed by mesenchymal cells. Further, the intra-tumoral heterogeneity of CAF populations with distinct phenotypes and functions have been described in murine and human tumors (44–46). This heterogeneity makes targeting CAFs as an anti-tumor treatment option more challenging.

The ECM consists of different matrix molecules including collagens, proteoglycans, hyaluronic acid and glycoproteins like laminins and elastin and increased levels of all these molecules have been correlated with poor prognosis (47–49). The most common feature of the ECM is high deposition of fibrillar collagen and upregulation of collagen processing enzymes, such as lysyl oxidase (LOX) (40). Secretion of LOX by tumor cells leads to cross linking of collagen and elastin and this highly crosslinked collagen matrix contributes to the extensive tumor rigidity and reduced oxygen supply to tumor (40, 50). Additionally, accumulation of proteoglycans and hyaluronic acid and their interactions with collagen within the tumor directly contribute to tumor stiffness by sequestering water (51, 52). As the tumor stiffens, there are mechanical forces that are generated from the surrounding healthy tissue pushing back on the growing tumor which induces solid stress (50). High solid stress can obstruct blood and lymphatic vessels leading to hypoxia and more interstitial fluid pressure which further acts to inhibit immune cell infiltration (53, 54). Altogether, the stiff ECM barrier prevents effective immune infiltration into the tumor core.

### 3.2 Hypoxia promotes the aberrant tumor vasculature and downregulation of adhesion molecules impairing immune cell infiltration

Other important factors that contribute to decreased intra-tumoral immune cell infiltration are tumor hypoxia and the aberrant tumor vasculature. Rapidly proliferating tumor cells have a high demand of oxygen, which is limited in the tumor. To support fast tumor growth, the tumor induces angiogenesis to increase nutrient and oxygen supply to the tumor. Hypoxia induces signalling of the major hypoxia inducible factor (HIF) pathway, which alters the balance in the production of pro- and anti-angiogenic factors promoting tumor angiogenesis (55). Specifically, activation of the hypoxia inducible transcription factor 1 (HIF-1) upregulates expression of vascular endothelial growth factor (VEGF) which can induce endothelial cell recruitment and blood vessel formation (55, 56). HIF-1 also upregulates MPPs, collagen modifying enzymes, and integrins which are all required for the formation of new blood vessels (56). Further, hypoxia and HIF-1α also induce production of angiopoietin-1, platelet-derived growth factor and TGF-β which drive vessel maturation through recruitment of supporting cells including pericytes to the newly formed vessels (57). Defective interactions between pericytes and endothelial cells can also contribute to tumor angiogenesis and formation of the disorganized neovasculature (58). For example, pericytes and endothelial cells are loosely associated in tumor capillaries in contrast to their tight associations in normal capillaries (59). Further, pericyte coverage on tumor vessels can vary from high to little or no coverage across different cancers (60). Low pericyte coverage can also result in increased vessel permeability, contributing to the vessel leakiness (60). The aberrant vasculature is also made up of anergic endothelial cells and is highly leaky leading to high interstitial fluid pressure causing vessels to collapse resulting in low perfusion into the tumor (61). Further, high levels of pro-angiogenic factors in the tumor can impair immune cell extravasation into the tumor site. Normally, immune cells including NK cells migrate towards an inflammation site through a chemokine gradient. Selectin-selectin interactions between NK cells and endothelial cells facilitates rolling along the endothelium (62). Chemokines present on the endothelium activate immune cells leading to integrin activation. The integrins, LFA-1 and VLA-4, expressed on the surface of NK cells bind to ICAM-1 and VCAM-1 respectively, facilitating firm interactions with the endothelium, followed by extravasation into the tissue (62). Increased levels of VEGF and bFGF can downregulate of expression of the adhesion molecules such as ICAM-1, VCMA-1 and E and P selectins, disrupting interactions with the endothelium (62). Overall, the resulting tumor vasculature is highly immature and disorganized, thus indirectly reducing infiltration of anti-tumor immune cells and promotes the immunosuppressive and hypoxic TME. Increases in pro-angiogenic factors due to hypoxic conditions induces lower immune cell and vessel wall interactions, also impairing immune cell infiltration into the tumor.

### 3.3 Chemokine receptor expression influencing NK cell tumoral infiltration

The two NK cell functional subsets express a variety of different chemokine receptors corresponding with their distribution within the body. While the CD56^bright^ subset is abundant within the liver, secondary lymphoid tissues, uterus, adrenal glands and the kidney, CD56^dim^ NK cells are prominent within the bone marrow, lung, spleen and breast tissue (48). The CD56^bright^ NK cells express CCR7 (CCL19 and CCL21 receptor), CCR5 (CCL3, CCL4, and CCL5 receptor), and the adhesion molecule CD62L (63, 64). The perforin and granzyme expressing CD56^dim^ subset express CX_3_CR1 (CX_3_CL1 receptor) (65). These CD56^dim^ NK cells also uniformly express CXCR1 and low levels of CXCR2 (CXCL1, CXCL2, CXCL3, CXCL5, and CXCL7 receptors) (66). In addition to chemokine receptors, CD56^dim^ NK cells also rely on sphingosine 1-phosphate (S1P) for trafficking (67). The CD56^bright^ and CD56^dim^ NK cells share expression of the CXCR4 (CXCL12 receptor) and CXCR3 (CXCL9, CXCL10 and CXCL11 receptor) chemokine receptors, however these are expressed to a lower extent in CD56^dim^ NK cells (68).

Various chemokine and chemokine receptors directly modulate NK cell homing towards solid tumors. Human clinical data demonstrates that solid tumors can upregulate chemokine expression which favors the recruitment of the CD56^bright^CD16- poorly cytotoxic NK cell subset. In turn, it also downregulates chemokines that recruit the CD56^dim^ cytotoxic subset (48, 69). Carrega et al. showed that in lung and breast cancer tissue, the percentage of CD56^bright^ and perforin^low^ expressing NK cells were much higher when compared to the corresponding normal tissue (70). This correlated with an upregulation of the chemokines CCL5, CCL19, CXCL9, and CXCL10 expression from the tumor (70). Hypoxic conditions have also shown to modulate chemokine receptor expression in peripheral blood NK cells. While IL-2 stimulation transiently enhanced CXCR4 expression, hypoxia significantly up-regulated CXCR4 expression on CD56^dim^ and CD56^bright^ NK cells compared to normoxic conditions (71). Intra-tumoral TGF-β can also modulate chemokine receptor expression. Active TGF-β1 present in the TME can upregulate expression of CXCR4 and CXCR3 in both CD56^bright^ and CD56 ^dim^ NK cells while also reducing CX_3_CR1 expression in CD56^dim^ NK cells (72, 73). Work in murine lymphoma tumor models has shown that the expression of CXCR3 and CX_3_CR1 chemokine ligands in the TME are needed for intra-tumoral accumulation of NK cells (74, 75).

Cytokine activation can also modulate chemokine receptor expression in human NK cells (76). The CX_3_CR1 receptor becomes downregulated in *ex vivo* activated human NK cells after cytokine stimulation with IL-15 (65). There is a general trend for downregulation of CXCR3 during short term stimulation with cytokines such as IL-2, IL-12, and IL-15 (69). Interestingly, the opposite has been seen with long term stimulation with IL-2 and K562 feeder cell-based NK cell expansion. Wennerberg et al. found that CXCR3 expression was significantly upregulated in *ex vivo* expanded NK cells (77). In addition, other reports show that while freshly isolated NK cells exhibited >80% expression of CXCR1, expansion of NK cells using K562 feeder cells expressing mb-IL21, mbIL-15 and 4-1BBL completely downregulated CXCR1 expression to undetectable levels (66). Expression of CXCR2 is also lost after NK cell *ex vivo* expansion using irradiated Epstein-Barr Virus lymphoblastoid cell lines as feeder cells (78). While human TNBC and renal cell carcinoma (RCC) cells express high levels of CXCR2 ligands including CXCL1 and CXCL2, a lack of CXCR2 expression by activated NK cells can limit their migration towards these tumors (78, 79). Expanded NK cells also downregulate expression of CXCR4 which is an important chemokine receptor for trafficking into the bone marrow (80). Further, expansion of NK cells can promote expression of chemokine receptors that promote NK cell trafficking to the liver including CXCR6 and CCR5 (80). Enhanced accumulation in the liver would diminish the number of NK cells that can enter non-hepatic tumor sites.

### 3.4 Maintaining potent NK cell anti-tumor functions within the solid tumor microenvironment

Even though NK cells may be able to infiltrate tumors, factors such as poor nutrient availability and immunosuppressive signals in the solid tumor microenvironment (TME) can inhibit NK cell tumor clearance. Thus, overcoming NK cell immune dysfunction within the TME is also necessary to sustain effective anti-tumor responses when targeting solid tumors. Apart from tumor and structural cells, various immune cells such as tumor associated macrophages (TAMs), regulatory T-cells (T-regs), myeloid derived suppressor cells (MDSCs) and neutrophils contribute to release of immunosuppressive factors such TGF-β and prostaglandin E_2_ (PGE_2_) that inhibit NK cell anti-tumor functions at the tumor site. Further, rapid tumor cell proliferation results in depletion of available nutrients in the TME which can impair NK cell function. Here we will summarize the inhibitory effects of TGF-β and PGE_2_ on NK cell function and discuss how NK cell expansion and cytokine activation can overcome inhibition in the TME.

In the TME, TAMs, T-regs, and MDSCs express soluble and membrane bound TGF-β while neutrophils can express the matrix metalloprotease (MMP)-9 to activate latent TGF-β to active TGF-β1 (81). TGF-β inhibits NK cell mediated killing by impairing the expression and signaling of NK cell activation receptors, primarily NKG2D. TGF-β directly reduces endogenous NKG2D expression in NK cells by inducing the production of microRNA(miRNA)-1245 which targets NKG2D transcripts (82). Furthermore, TGF-β can decrease the expression of DAP10 at the mRNA and protein level, impairing the intracellular signaling cascade of NKG2D (83). Lastly, TGF-β can induce the expression of miR-183 which promotes the downregulation of DAP12 and impairs the signal transduction of the activating receptor NKp44 (84). Thus, TGF-β renders NK cells unable to recognize malignant cells effectively by impairing the signal transduction of their activation receptors. TGF-β can also inhibit NK cell function by reducing their metabolic capacity. In mouse NK cells, *in vitro* culturing with TGF-β can block the induction of the mTOR pathway by IL-15 and decreases granzyme B and perforin expression following IL-2 stimulation (85). TGF-β also significantly decreases IL-2 induced mitochondrial metabolism in human NK cells. It inhibits the rates of oxidative phosphorylation and maximal respiration, while having no effect on glycolytic basal rate but reducing the glycolytic capacity of the cells (86). This impaired metabolic activity correlates with decreased IFN-γ, granzyme B and TRAIL expression on NK cells. Interestingly, this effect was found to occur without affecting mTORC1 signaling as seen in murine NK cells (86). Therapies inhibiting TGF-β are currently in development and are being evaluated for clinical efficacy and safety. Treatments blocking the synthesis of TGF-β and TGF-β receptor 1 kinase inhibitors have shown promise in high-grade gliomas, while a small molecule TGF-β1 inhibitor is demonstrating promising efficacy in the treatment of HCC (87, 88). It would be important to determine if rescued NK cell activity is playing a role in the efficacy of targeting intra-tumoral TGF-β. However, one study has demonstrated that the anti-tumor function of tumor-infiltrating NK cells isolated from patients with glioblastoma multiforme could not be rescued with administration of a TGF-β receptor kinase inhibitor (89). These authors demonstrated that genetic deletion of TGF-β on NK cells significantly improved tumor control *in vivo (* 89).

PGE_2_ is another factor that can impair NK cell function within the TME. The expression of PGE_2_ in human gastric and prostate cancer has been inversely correlated with overall patient survival (90, 91). Cancer cells and CAFs produce PGE_2_ through upregulation of cyclooxygenases COX1 and COX2 (92). The receptors of PGE_2_ (EP1-EP4) are present on multiple cell types and murine NK cells express all four PGE_2_ receptors (93). Signalling of PGE_2_ through the EP4 receptor is primarily responsible for the dysfunction of murine NK cells leading to the inhibition of IFN-γ and TNF-α. Blocking the EP4 receptor with a small molecule can rescue murine NK cell activity *in vivo (* 94). In human NK cells, the EP2 and EP4 receptors are responsible for the suppression of human NK cell cytotoxicity mediated through NKG2D, CD16, and NCRs (95, 96). PGE_2_ can act through these receptors to inhibit the activation of the ERK and NF-κB pathways on NK cells, thus leading to a reduction in their cytotoxic activity and IFN-γ production (95). PGE_2_ can also act on human type 1 conventional dendritic cells (DCs) leading to anti-tumor dysfunction by downregulating their expression of T cell recruiting chemokine ligands such as CXCL9 and CXCL10 and production of IL-12 which could also impair NK cell function (97). Initial therapies targeting COX-2 in tumours to reduce the expression of PGE_2_ in the TME were effective in arresting tumour growth, however, the off-target inhibition of COX-1 caused serious adverse side effects affecting the gastro-intestinal and cardiovascular system (98). Current research efforts are focusing on developing a COX-2 specific inhibitor that would avoid any of the side effects related with COX-1 inhibition and could be beneficial in combination with NK cell-based immunotherapies. EP antagonists could also be combined with NK cell adoptive cell therapy to sustain NK cell function in the TME.

Tumor resistance to NK cell therapy can also be attributed to the immense metabolic requirements of rapidly proliferating tumor cells resulting in poor nutrient availability in the TME (99). Indeed, maintaining high metabolic fitness in the TME is necessary to sustain high CAR-NK cell function and prevent tumor escape (99). We have shown that expanding NK cells using K562 feeder cells expressing mb-IL-21 reprograms NK cells towards a metabolically flexible phenotype, which not only maintains, but also enhances their cytotoxicity within a hostile and nutrient deprived TME (100). Further arming CAR-NK cells with activating cytokines like IL-15 can enhance their *in vivo* persistence and improve their metabolic fitness, leading to enhanced tumor control (99). However, CAR-NK cells expressing IL-15 eventually succumbed to immune dysfunction losing their ability to kill target cells by day 35 post administration compared to the pre-infused product (99). This loss in long-term metabolic fitness may necessitate multiple CAR-NK cells doses to ensure durable responses (99). Further, deletion of the cytokine-inducible SH2-containing protein (CIS) in cord blood-derived CAR-NK cells expressing IL-15 significantly enhances their persistence and boosts their metabolic fitness (101). CIS acts as a negative regulator of cytokine signaling including IL-2 and IL-15 signaling and is an important checkpoint in NK cells (102). Knockout of the gene encoding for CIS (*CISH)* lowers the activation threshold of NK cells and enhances NK cell sensitivity to IL-15 improving their anti-tumor function (102). *CISH* knockout in iPSC-derived NK cells expanded with K562 mb-IL-21 feeder cells also enhances their metabolic fitness and persistence (103). Whether further engineering CAR-NK cells with a CISH knockout will sustain their long-term metabolic fitness within the TME in a clinical setting remains to be determined. Overall, providing IL-15 signaling may be necessary to maintain NK cell persistence, metabolic fitness, and enhanced anti-tumor functions in the TME. **Table 1** summarizes the barriers posed by the solid tumor which prevent efficient NK cell infiltration and activation.

**Table 1:** Summary of barriers posed by the solid tumor that hinder NK cell infiltration and activation in the tumor site.

Type of Tumor Barrier	Cells or molecule(s) involved	Role/effect of cells or molecule(s)	Overall effect on tumor	Overall effect on NK cells
**Extracellular Matrix**	Tumor cells	• produce collagen I and collagen III • produce ECM-modifying enzymes including lysyl oxidase (41) • induce stromal cell differentiation into CAFs (40)	• high solid stress • increased tumor stiffness • higher vessel compression • reduced oxygen supply to tumor • higher interstitial fluid pressure • EMT promotes tumor cell invasion through basement membrane leading to metastasis	• impaired tumor infiltration
	CAFs	• produce collagen and TGF-β (40) • drive epithelial to mesenchymal transition (EMT) (40)		
	Collagen and proteoglycan	• proteoglycans interact with collagen • collagen crosslinks with elastin contributing to tumor rigidity (51, 52)		
	Hyaluronic acid	• interacts with collagen and proteoglycans and sequesters water leading to tumor stiffness (51, 52)		
	Lysyl oxidase	• crosslinks collagen and elastin (40, 50)		
Aberrant vasculature	Hypoxia inducible transcription factor	• induces production of VEGF and basic fibroblast growth factor (55, 56)	• higher vascular permeability • low blood perfusion	
	VGEF and basic fibroblast growth factor	• promotes endothelial cell recruitment • promotes vessel formation (55, 56) • lowers adhesion molecules on endothelial cells (ICAM-1, VCAM-1, E and P-selectins) (62)		
	Angiopoetin-1 and platelet derived growth factor	• recruit pericytes to promote vessel maturation (58)		
	Pericytes	• low pericyte coverage and loose association with tumor capillaries leads to vessel leakiness (58)		
	MMPs, collagen modifying enzymes, integrins	• required for new vessel formation (56)		
	Anergic endothelial cells	• contributes to collapsed and narrow vessels (61)		
Immunosuppression	TGF-β	• targets NKG2D transcripts (82) • decreases expression of DAP10 and DAP12 (83) • inhibits human NK cell oxidative phosphorylation and NK cell glycolytic capacity (86) • upregulates expression of CXCR4 and CXCR3 in CD56^bright^ and CD56^dim^ NK cells (72, 73) • reduces expression of CX_3_CR1 in CD56^dim^ NK cells (72, 73)	• early-stage malignancy: reduced cell proliferation and differentiation, induces cell apoptosis and DNA damage (104) • late-stage malignancy: induces EMT, cell proliferation, angiogenesis, and metastasis (104)	• reduced NKG2D expression and signaling (82) • reduced NKp44 (83) signaling • decreased metabolism leading to lower IFN-γ, granzyme B and TRAIL expression (86) • higher tumor infiltration of low cytotoxic CD56^bright^ NK cells (72, 73)
	PGE_2_	• impairs DC-derived IL-12 and CXCL9/CXCL10 production (97) • inhibits ERK and NFkB signaling in NK cells (95) • promotes stimulation of VEGF (92)	• enhances tumor cell proliferation and survival (92) • promotes angiogenesis and enhances vascular permeability (92)	• reduced IFN-γ expression • reduced NKG2D expression • reduced cytotoxicity (95, 96)

## 4 Strategies to enhance NK cell homing and infiltration into the solid tumor

**Figure 1:**
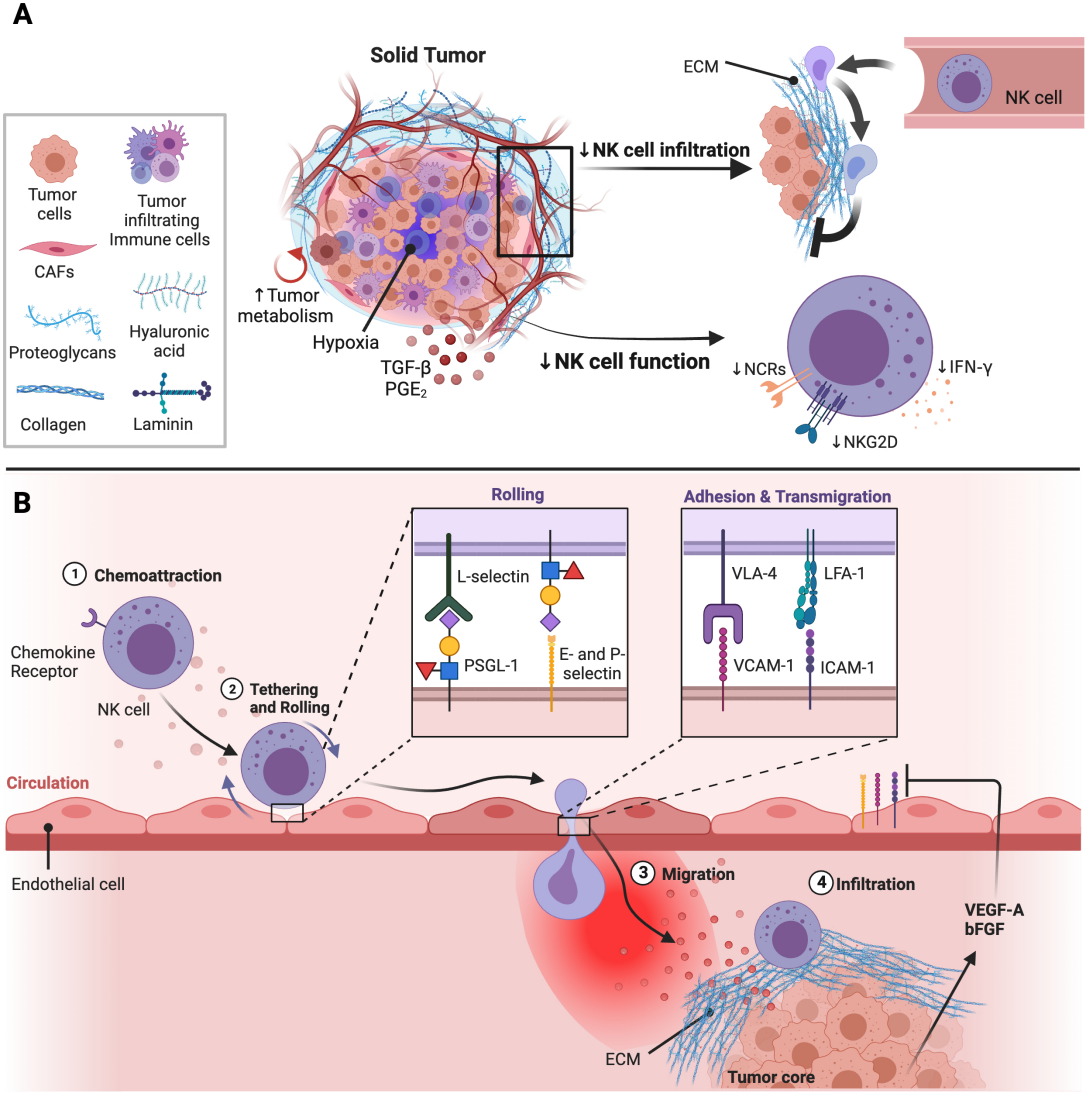
Impact of the TME on NK cell function and infiltration into solid tumor sites. **(A)** The solid tumor consists of tumor cells, immunosuppressive immune cells, and cancer associated fibroblasts (CAFs) which inhibit NK cell function and infiltration. CAFs are major producers of components that make the extracellular matrix (ECM). The ECM is made up different molecules including collagen, proteoglycans, hyaluronic acid, and laminin which contribute to tumor stiffness and solid stress. Solid stress generated can obstruct the tumor vasculature leading to hypoxic conditions in the TME. Further, the high energy demands of rapidly proliferating tumor cells leads to poor nutrient availability in the TME decreasing NK cell metabolic fitness and anti-tumor activity. Lastly, immunosuppressive factors enriched in the TME such as TGF-β and PGE_2_ also inhibit NK cell function through downregulation of activation receptor expression and signaling. Adapted from “Tumor Extracellular Matrix Reduces Therapeutic Efficiency in Solid Tumors”, by BioRender.com (2023). Retrieved from https://app.biorender.com/biorender-templates. **(B)** Production of pro-angiogenic factors including vascular endothelial growth factor (VEGF)-A and basic fibroblast growth factor (bFGF) induced by hypoxic conditions within the TME can downregulate expression of adhesion molecules on endothelial cells impairing NK cell extravasation into tumor sites. After NK cells migrate towards the tumor, NK cells are shielded from tumor cells by the dense and stiff ECM surrounding the tumor core. Adapted from “Leukocyte Extravasation - The Role of Glycans in Inflammation”, by BioRender.com (2023). Retrieved from https://app.biorender.com/biorender-templates.

As summarized in **Figure 1**, the aberrant tumor vasculature is highly leaky while the solid stress generated by the growing tumor can further compress the tumor vasculature reducing blood flow into the tumor bed. This enhances hypoxic conditions and pro-angiogenic factors leading to less immune cell interactions with the vasculature. Further, the rigid ECM barrier prevents immune cell entry into the tumor core, inhibiting direct interactions with tumor cells. These factors can negatively impact delivery of anti-cancer drugs and infiltration of immune effector cells into solid tumors. To overcome this, various anti-stroma and vascular normalization strategies have been developed to enhance blood perfusion throughout the solid tumor core. In addition, genetic engineering of NK cells to express chemokine receptors or altering chemokine expression within the TME is being actively pursued.

### 4.1 Breaking down the ECM and normalizing the tumor vasculature to promote immune cell tumor infiltration

Directly targeting the tumor stroma can reduce ECM deposition and solid stress of the tumor. One such strategy involves the use of Angiotensin II receptor blockers (ARBs). ARBs are common anti-hypertensive drugs, but their anti-tumor effects have also been investigated as angiotensin II has been shown to contribute to tumor ECM formation (105). Fibroblasts such as CAFs also express the angiotensin II receptor type I (ARTI) (106). Further, patients with ovarian cancer exhibit elevated levels of the angiotensin converting enzymes which converts angiotensin I to the bioactive angiotensin II (107). In ovarian and breast cancer murine models, blocking ARTI signaling can downregulate collagen I expression, decompress vessels, increase vessel perfusion, and reduce solid stress (108, 109). This strategy can be used to enhance intra-tumoral delivery of other anti-cancer drugs such as chemotherapies. Specifically, Zhao et al. have demonstrated that while stroma normalization with Losartan, an FDA approved ARB, did not reduce tumor burden in human ovarian cancer models it did enhance the anti-tumor effects of the chemotherapy agent paclitaxel when used in combination (109). Here, combination therapy led to a significant reduction in the incidence and volume of ascites in the tumor bearing mice (109). Similarly, using a tumor selective ARB alone had no anti-tumor efficacy but when combined with checkpoint immune blockade there was a 66% response rate in a TNBC syngeneic mouse model (108). In addition, this combination therapy increased the number of intra-tumoral CD45+ immune cells and CD8+ cytotoxic T cells (108). Further, ARBs can also modulate chemokine expression in the TME. Nakamura et al. found that ARB treatment significantly lowered the expression of CXCL12 by ARTI-expressing CAFs in a murine model of colon cancer (110). CXCL12 is expressed in a variety of tumors including ovarian cancer, breast cancer, glioblastoma and pancreatic cancer (111). CXCL12 has been demonstrated to promote tumor cell proliferation and survival and can synergize with VEGF to induce neoangiogenesis *in vivo (* 111–113). High levels of CXCL12 in the tumor can attract CXCR4-expressing immune cells, fibroblasts and endothelial cells to the tumor site further promote tumor growth (113). Recruitment of FAP-expressing CAFs via CXCR4/CXCL12 signaling can prevent accumulation of intra-tumoral cytotoxic T cells (114). Hence, targeting the CXCR4/CXCL12 axis to promote immune cell infiltration within tumors has been of interest. Chen et al. directly targeted the CXCR4/CXCL12 signaling axis in metastatic breast cancer *in vivo* models with the FDA approved drug plerixafor (115). They found that blocking the CXCR4 receptor expressed on α-SMA+ cells including CAF cells and pericytes led to decreased tumor fibrosis, less solid stress, higher degree of decompressed blood vessels and enhanced cytotoxic T cell infiltration (115). Similarly, remodeling of the stroma and vasculature also doubled the response to immune checkpoint blockade therapy (115). Targeting the ECM by administration of MMP inhibitors or enzymes that breakdown ECM components such as hyaluronan have also been tested with low success (116). A clinical trial with chemotherapy and pegylated recombinant human hyaluronidase combination treatment showed decreased overall survival in patients with metastatic pancreatic cancer due to treatment induced toxicity (117). Further, altering hyaluronan could also enhance the glucose metabolism of tumor cells by increasing their expression of the Glut1 glucose transporter, promoting their migration capacity (118). Directly targeting the tumor stroma through CAR-T cells is another strategy that has been investigated. Depletion of FAP-expressing CAF cells by CAR-T cells enhanced intra-tumoral CD8+ T cells and FN-γ production significantly impeding tumor growth (119). However, a similar strategy has demonstrated that directly targeting FAP-positive cells can lead to lethal bone marrow toxicity and muscle loss due to the expression of FAP in multipotent bone marrow stromal cells (120). Further, Xie et al. have shown that targeting the EIIB fibronectin splice variant which is mainly expressed in the tumor ECM and neovasculature can also delay tumor growth and improve survival in melanoma tumors (121). Directly targeting CAFs could also improve migration of immune cells to the tumor core by modulation of the CXCL10/CXCL9-CXCR3 axis. Primary CAFs isolated from pancreatic cancer samples have been shown to downregulate the expression of CXCR3 on human T cells leading to reduced T cell migratory capacity towards CXCL10 *in vitro (* 122). Depletion of CAFs could enhance CXCR3 ligand availability in the TME. However, complete depletion of the tumor stromal components such as CAFs may not be feasible anti-tumor strategy due to possible toxicities and unwanted side effects. Instead, strategies that modulate the functions of specific CAF populations could lead to improved therapeutic responses. For example, TGF-β blockade has shown to reduce expression of genes involved in ECM and induced an IFN-response gene signature in murine and human CAFs, including upregulation of CXCR3 ligand expression (45). TGF-β blockade also led to a significant increase of tumor infiltrating cytotoxic T cells in a murine breast cancer model (45).

Vascular normalization strategies aim to remodel the highly disorganized tumor vasculature to resemble the more mature normal vessels which have higher pericyte coverage to improve blood perfusion (123). While the use of anti-angiogenic molecules such as VEGFR inhibitors have been widely investigated for their anti-tumor effects, monotherapy with angiogenic inhibitors has not yielded much clinical success (124, 125). Further, careful considerations for the dosing and timing of anti-angiogenic therapy are critical to avoid excessive vascular death which could prevent perfusion and further enhance hypoxia (126). Huang et al. demonstrated that a low dose of an antibody blocking the VGEF receptor (VGEFR), VGEFR2, combined with a cancer cell vaccine significantly inhibited the tumor growth in breast cancer models (127). Administration of the anti-VGEFR2 therapy led to higher perfusion of the tumor and improved distribution of perfused vessels. They also found significantly higher levels of infiltrating CD4+ and CD8+ T cells when combined with a vaccine (127). Another study utilizing dual PD-1 and VEGFR2 blockade significantly inhibited tumor growth and doubled survival in an orthotopic mouse model of hepatocellular carcinoma (128). The authors demonstrated that combination therapy increased the number of pericyte covered vessels and enhanced micro vessel density. They also noted an enhancement CD8+ T cell infiltration and downregulation of the M2 pro-tumor macrophages and T-regs (128). Interestingly, immunotherapy and checkpoint blockade therapies alone has also shown to promote tumor vessel normalization. Tian et al. showed that adoptive transfer of type 1 T helper cells (Th1) in a patient derived xenograft TNBC tumor model improved tumor perfusion and reduced vessel leakiness and hypoxia (129). Monotherapy with immune checkpoint blockade has been demonstrated to increase tumor vessel perfusion in multiple breast cancer mouse models through the enhanced expression of intra-tumor IFN-γ (130). IFN-γ acts on vessels to induce blood vessel death and can inhibit formation of new blood vessels through downregulation of VEGF (131, 132). Production of high levels of IFN-γ in the TME by expanded NK cells could act as a positive feedback loop to promote vascular normalization and further enhance immune infiltration. Although these studies have not directly examined whether stroma or vascular normalization can specifically enhance NK cell tumor infiltration, they provide evidence that therapies that enhance tumor perfusion could synergize with NK cell adoptive cell therapies to improve entry into the tumor bed. There is currently one phase II clinical trial in recruitment that will be assessing the efficacy and safety of a VGEFR2 tyrosine kinase inhibitor (Apatinib) and an anti-PD-1 (Pembrolizumab) antibody in combination with cord blood-derived NK cells for HCC (NCT05171309).

### 4.2 Directly modulating chemokine-chemokine receptor expression to enhance NK cell migration

Modulating chemokine expression within the tumor or altering the expression of chemokine receptors in NK cells can influence their migration into solid tumor sites. One strategy involves genetic engineering of NK cells to express chemokine receptors that correspond to the specific chemokines present within the tumor being targeted. For example, engineering the YTS NK cell line to co-express an anti-EGFRvIII CAR and CXCR4 led to enhanced NK cell infiltration and improved survival in glioblastoma xenograft models overexpressing CXCL12 (133). Similarly, human *ex vivo* expanded NK cells have also been engineered to co-express CXCR4 with a gain-of-function mutation and an anti-BMCA CAR using mRNA electroporation for treatment of multiple myeloma (134). While there was enhanced NK cell migration to the bone marrow and tumor control, the treatment did not completely abolish tumors leading to relapse which could be overcome through stable expression of the CAR (134). The human NK cell line, NK-92s, have also been engineered to overexpress CXCR4 and CCR7 to promote their migration in human colon cancer *in vivo* models (135). Other chemokine receptors have also been expressed on NK cells to target different tumors. For example, expression of CXCR2 in human NK cells using retroviral transduction significantly enhanced their migration towards CXCR2 recombinant ligands and RCC lines *in vitro (* 78). Further, enhanced migration of CXCR2-expressing NK cells led to higher killing of target cells compared to the NK cells transduced with the control vector (78). Another study showed that overexpression of CXCR1 via electroporation significantly enhanced human expanded NK cell infiltration in a human HNSCC xenograft model (66). Specifically, using NIR fluorescently labeled NK cells they found that after 72h post intravenous injection the CXCR1-expressing NK cells localized to the subcutaneous tumor sites with a 10-fold increase in signal intensity compared to mock NK cells. Additionally, NK cells co-transfected with CXCR1 and an NKG2D-CAR significantly improved survival in a human ovarian cancer model (66). Another viable strategy to improve NK cell infiltration to tumor sites involves the downregulation of chemokine receptors that are involved in NK cells trafficking to the liver. For example, Levy et al. demonstrated that downregulating CCR5 expression on expanded NK cells using CRISPR/Cas9 significantly reduced homing to the liver after intravenous administration (80). Combining the deletion of CCR5 with overexpression of other chemokine receptors could enhance NK cell infiltration away from the liver and into the targeted tumor site. The concentration of chemokines within the TME can also be altered to improve NK cell infiltration. For example, administration of an irradiated tumor vaccine secreting CCL3 led to a 3-fold increase in the number of NK cells within murine CT26 colon tumors (136). Accumulation of NK cells within the tumors contributed to slowed tumor growth and augmented T cell infiltration through induction of CXCL9 and CXCL10 via NK cell-derived IFN-γ (136).

Other strategies which do not rely on genetic engineering of NK cells with chemokine receptors to improve infiltration of NK cells in the tumor have also been investigated. One novel strategy involves the use of an NK cell recruiting protein-conjugated antibody (NRP-body) that can be selectively activated within the TME. Lee et al. demonstrated that an NRP-body specific for mesothelin and conjugated with a cleavable soluble form of CXCL16 is released in the local tumor environment through cleavage by furins that are highly expressed on the surface pancreatic cancer cells (137). This NRP-body significantly enhanced the infiltration of human expanded NK cells to primary and metastatic tumor sites in a preclinical pancreatic ductal cell carcinoma mouse model (137). In glioblastoma cancer models, inhibition of the b-galactoside-binding lectin, galectin-1 has shown to enhance expression of CXCL10, CXCL12 and CCL5 resulting in recruitment of Gr-1+/CD11b+/CCR2+ myeloid cells which promoted the recruitment of tumor-infiltrating NK cells (138).

Although the impact of NK cell intra-tumoral infiltration with stroma or vascular normalization therapies has not been thoroughly investigated, enhanced perfusion to the tumor by reducing vessel compression has the potential to improve adoptive NK cell immunotherapy. Further, strategies that directly modulate the chemokine/chemokine receptor axis have shown to specifically enhance NK cell trafficking and infiltration to tumor sites in various tumor models.

## 5 Strategies for tumor-targeted delivery of immunomodulatory agents to pull and boost NK cells in the TME

**Figure 2:**
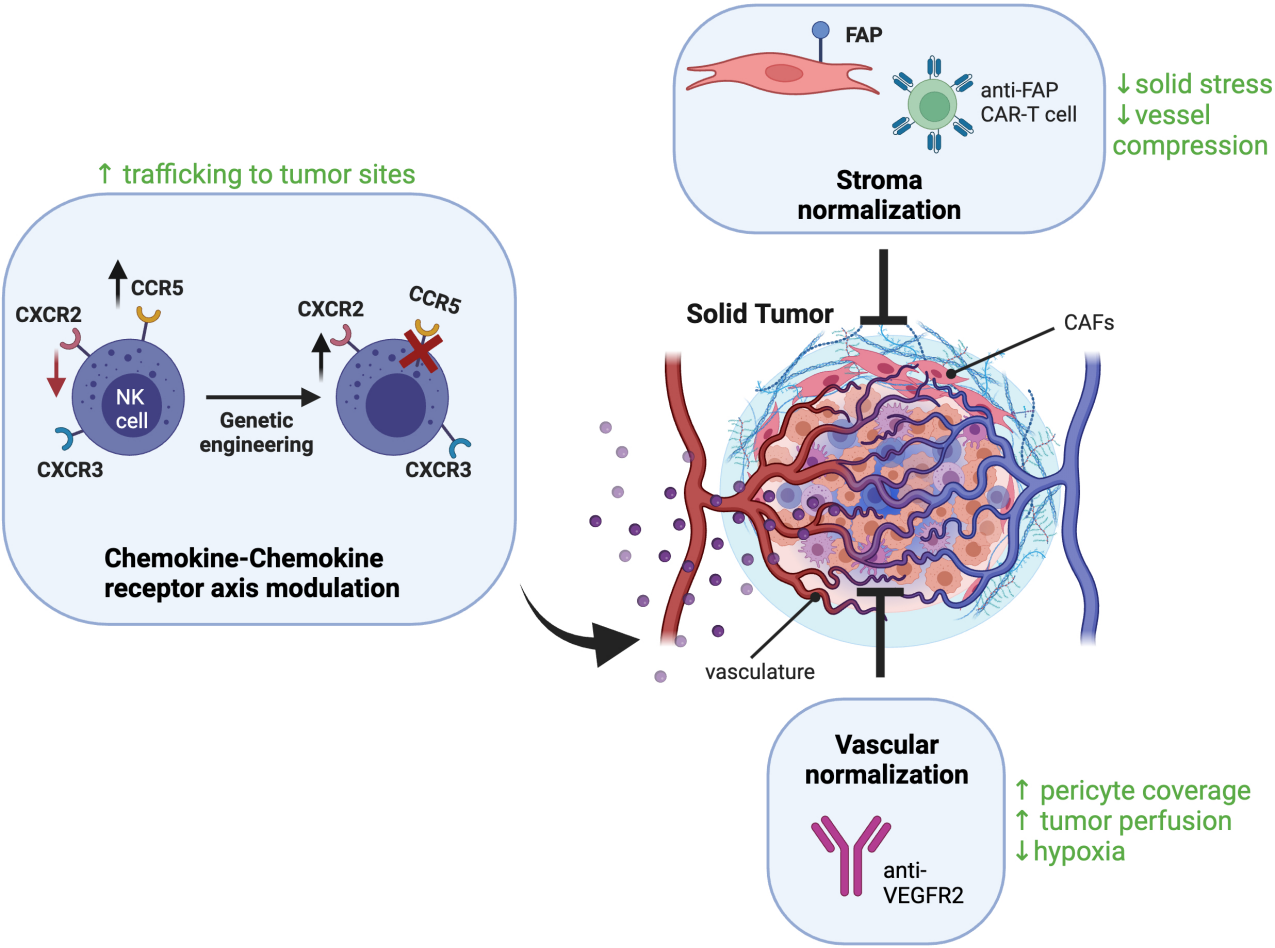
Strategies to enhance NK cell infiltration into solid tumors. Different CAF‐targeting strategies are being explored for stroma normalization including elimination of CAFs using CAR-T cells against CAF surface markers like fibroblast activation protein (FAP). These strategies can relive solid stress of the tumor, reduce ECM formation, and reduce vessel compression to improve tumor perfusion. Vascular normalization strategies aim to improve tumor perfusion by reducing vessel formation and increasing pericyte recruitment, one example is blockade of the VEGF receptor VEGFR2. To enhance NK cell trafficking to the tumor site, NK cells can be engineered to overexpress chemokine receptors corresponding to chemokines expressed by the tumor. Created with BioRender.com.

While the strategies discussed above and summarized in **Figure 2** are promising, there are limitations that can preclude their efficacy including: 1) potential unwanted side effects associated with systemic administration of anti-stroma and anti-angiogenic strategies 2) modulating NK cells based on the chemokines endogenously expressed by specific tumors would limit the therapeutic potential of these strategies across various tumor types. Alternatively, tumor-targeted delivery of agents that promote stroma and vascular normalization as well as recruit and activate NK cells to the tumor site would address some of these limitations. Oncolytic viruses (OVs) are an attractive platform for combination therapy with NK cells given their ability to selectively replicate in a wide range of tumor types and their capacity to express a wide range of immunomodulatory agents (139). In contrast to strategies discussed in the previous section, engineered OVs have the added advantage of limiting the effects of the payload to the tumor site. In this section we summarize how OVs can be used to enhance NK cell infiltration and activation in the TME. We also discuss the challenges of applying OVs for cancer therapy and discuss other methods available to deliver immunomodulatory therapies to the TME.

### 5.1 Oncolytic viruses trigger direct tumor oncolysis and induce NK cell recruitment and activation

Oncolytic virotherapy for cancer treatment harnesses the ability of OVs to effectively infect and replicate in cancer cells. This is attributed to the fact that unlike healthy cells, cancer cells have dysregulated IFN signaling, which typically activates host antiviral responses to shut down virus replication (140). OVs subsequently exert direct anti-tumor activity through direct oncolysis and through the stimulation of innate and adaptive anti-tumor immunity (141). A variety of different OVs have been investigated for oncolytic virotherapy, including herpes simplex virus (HSV), adenoviruses, vesicular stomatitis virus (VSV), measles virus (MV), vaccinia virus (VV), and Newcastle Disease virus (NDV) (140). Currently, the only fully US Food and Drug Administration approved oncolytic virotherapy is talimogene laherparepvec (T-VEC), while there have been three other OVs approved around the world (142, 143). T-VEC is an engineered OV derived from HSV-1 expressing human granulocyte macrophage colony-stimulating factor (GM-CSF) used to treat unresectable melanoma (142). However, other OVs are currently being explored in clinical trials for a wide variety of solid tumors.

While OVs can trigger direct lysis of infected tumor cells, their primary anti-tumor function is maintained by their ability to elicit high levels of inflammatory immune responses. OVs can induce tumor cell death that can stimulate an immune response, termed immunogenic cell death (ICD). After OV replication and oncolysis, dying cancer cells undergo cell death pathways which cause the release of virus-derived pathogen-associated molecular patterns (PAMPs) and cell host-derived damage-associated molecular patterns (DAMPs) (141). DAMPs typically induced by ICD include the release of cellular contents such as ATP high-mobility group box 1 (HMGB1) and heat shock proteins (HSP) such as HSP90 (144). OVs are thought to activate type II ICD, which is characterized by induction of immunogenic apoptosis through targeting of the ER (144). Each OV has a unique ability to induce ICD based on its ability to infect and lyse tumor cells (145). OV infection leads to the local release of type 1 IFNs and chemokines, which attract DCs to the tumor site and enhance their expression of MHC molecules and the uptake of tumor cell debris which promotes recruitment and expansion of anti-tumor lymphocytes (146). In addition, mature DCs produce IL-12, IL-18, and type I IFNs to activate NK cells (147). The pro-inflammatory environment and immune cell-attracting chemokines generated at the tumor site promotes the trafficking of activated NK cells and tumor-specific CD8+ T cells (146). Additionally, NK cell-derived chemokines including CCL5 and XCL1 can also mediated recruitment of DCs into the tumor leading to improved tumor control in murine solid tumor models (148). The ability of OVs to promote robust immune mediated anti-tumor responses is critical to achieve sustained anti-tumor efficacy with studies suggesting that the immunogenicity of an OV is more important than early replication in tumors, and that their efficacy is primarily due to lymphocyte infiltration and recruitment to the tumor. This allows them to be leveraged as candidates for combination with adoptive cell therapies, as they can promote homing of adoptively transferred immune cells and enhance their activation.

Several studies demonstrate that OVs have the capacity to activate and enhance NK cell infiltration in various cancer models. For example, administration of one to five doses of oncolytic reovirus to patients with colorectal cancer led to type 1 IFN production and NK cell activation, marked by elevated expression of the activation marker CD69 on these cells (149). In addition, infection of TNBC cells by VSV virus can enhance expression of immune cell attracting chemokines, leading to enhanced migration of NK cells *in vitro (* 150). OV infection of tumors themselves also induces stress ligand expression on tumor cells as a mechanism of conventional antiviral defense to elicit successful NK cell responses, enhancing NK cell activation. Work has shown that the immunogenic effect of OVs can be enhanced by using secondary drug therapeutics, such as histone deacetylase inhibitors, to enhance the extent of the inflammatory response. Indeed, combining the HDACi valproic acid with T-VEC enhances GM-CSF production induced by the virus, and as well enhances tumor killing by increasing expression of NK cell activating ligands and endogenous tumor antigens on tumor cells (151). Additionally, various studies using Orf virus (OrfV) as an oncolytic agent have demonstrated that its anti-tumor efficacy relies heavily on NK cells (152, 153). OrfV is a dsDNA virus of the *Parapoxvirus* genus that naturally infects sheep and goats but causes mild infection in humans (154). Work in metastatic lung tumor models have shown that OrfV can significantly reduce lung metastasis formation in a mechanism dependent on NK cells (153). Further, OrfV administration significantly enhanced the proportion of IFN-γ and granzyme positive NK cells in the spleen, blood, and lungs of OrfV treated mice (153). In a study analyzing postoperative NK cell dysfunction, preoperative administration of OrfV significantly reduced formation of lung nodules in a 4T1 mammary carcinoma tumor model and enhanced the cytotoxic function of these dysfunctional NK cells (155). OrfV has also been used in a murine end-stage ovarian cancer model showing efficacy as a monotherapy through stimulation of NK cell responses leading to enhanced recruitment of CD8+ CXCR3-expressing T cells through upregulation of CXCR3 ligands (156). The significant reliance of OrfV on NK cells makes it an attractive OV to engineer to enhance NK cell infiltration into tumor sites. Moreover, since OrfV primarily infects sheep there is likely to be an existing immune response against the virus that could shut down its ability to infect tumor cells which is the case with other OVs such as adenoviruses (157). However, OrfV has a limited tumor tropism with low replication capacity in various human cancer cells which would hinder its clinical use against a wide range of cancer types (152, 153).

### 5.2 Engineering oncolytic viruses to enhance NK cell tumor infiltration and function in TME

OVs can also be engineered to express exogenous immunostimulatory molecules to enhance NK cell activation and function in the immunosuppressive TME. In a murine breast cancer model, combination therapy with VV and a 4-1BB agonist significantly increased levels of intra-tumoral NK cells by nine days post administration and depletion of NK cells reduced therapeutic efficacy of the treatment (158). Administration of VV expressing a murine IL-15 and IL15 receptor α (IL-15/IL-Rα) complex induced gene expression of NKp46 and NKG2D in murine ovarian cancer tumors suggesting enhanced NK cell infiltration in the tumor (159). However, depletion of NK cells had no impact on tumor progression or survival in this study. Co-expression of IL-12 and IL-15/IL-Rα using oHSV-1 has also been demonstrated to promote efficient tumor clearance in syngeneic colon and sarcoma models (160). This strategy resulted in a slight, albeit not significant, enhancement in the percentage of NK cells in treated CT26 tumors (160). There are currently still very limited pre-clinical studies employing the combination of OVs and NK cell adoptive cell therapy for solid tumors. A previous study utilizing VV expressing the CCL5 chemokine was used to promote NK cell migration leading to over 50% complete response rates in human colon cancer models (161). However, this effect was only seen when mice were also administered engineered CCR5-expressing NK cells (161). Other studies have used oHSV-1 and CAR-NK cell combination therapies for the treatment of glioblastoma (162, 163). Administration of oHSV-1 expressing the IL-15/IL-Rα complex (OV-IL15C) and primary anti-EGFR CAR-NK cells led to significantly longer survival than the monotherapies alone (163). Further, OV-IL15C increased persistence of the EGFR CAR-NK cells in the brain compared to EGFR CAR-NK cells alone, yet they did not exhibit an exhausted phenotype within the TME (163). This suggests that OVs can be used for both immune cell recruitment to solid tumors, while also enhancing their survival and cytotoxic function once within the tumor. While neurotoxicity that results from inflammation during OV infection is a concern, new work in humans has shown that direct administration of an oncolytic adenovirus into the brain can prolong survival with limited toxicity (164). OVs can also be engineered to express other immunostimulatory molecules to stimulate intra-tumoral NK cells as a combination therapy. A recent study demonstrated that OVs can redirect and activate NK cells towards the tumor through expression of a bispecific killer engager (BiKE) which possess a tumor antigen binding domain and NK cell binding domain. Floerchinger et al. generated a BiKE-encoding MV and found that tumors infected with the MV-BiKE construct significantly enhanced the degranulation capacity of human NK cells present in the patient-derived pancreatic cancer tumor tissue (165). Further, MV-BiKE treatment was also able to enhance the degranulation, IFN-γ production and killing capacity of allogenic human NK cells in a co-culture model with primary pancreatic cancer cells. However, the enhanced killing varied and was dependent on the degree of permissiveness of the patient tumor sample to MV infection (165). Other immunomodulatory factors that could be expressed by OVs to activate NK cells include the bifunctional TGF-βRII and IL-15/IL-15Ralpha protein domain which has been shown to promote NK cell infiltration into murine melanoma tumors (166). A recent study has also shown that VV can be engineered to express a TGF-βRII inhibitor to outcompete local TGF-β in the TME (167). This engineered OV rescued anti-tumor efficacy and significantly increased survival in a murine melanoma tumor model resistant to wild type VV treatment (167). This anti-tumor effect was correlated with a less suppressive T-reg phenotype but could also synergize with other immune modalities sensitive to TGF-β inhibition such as NK cells. Several OVs that are similarly engineered with cytokines and other recombinant proteins are currently in early clinical trials as therapies for numerous types of solid tumors (**Table 2**). Common candidates for engineering include IL-12, IL-15, IL-21, type 1 IFN, and other immune stimulatory agents such as 4-1BBL. Many of these approaches combine cytokine expressing OVs with checkpoint inhibitors, that increase lymphocyte infiltration and activity within the TME, and a few also combine these OVs with T cell therapies. Overall, co-expression of these cytokines or other activating molecules that can specifically boost NK cell anti-tumor activity and chemoattracting factors will be advantageous when considering OVs and NK cell combination therapies.

**Table 2:** Clinical trials using engineered oncolytic viruses to enhance immune recruitment and activation for solid tumor therapy.

	Trial ID	Malignancy	Phase	Transgene	Combination therapeutic	Intended mechanism of action
Vaccinia Virus	NCT05914376	Advanced solid tumors	1	IL-21	–	Immune activation
	NCT02017678	Ovarian carcinoma	2	GM-CSF	–	Immune recruitment
	NCT04301011	Advanced solid tumors	2	IL-12, α-CTLA-4	Pembrolizumab	Immune recruitment and evasion of immunosuppression
Adenovirus	NCT02045589	Pancreatic adenocarcinoma	1	PH20 hyaluronidase	Gemcitabine, Abraxane	ECM degradation for improved drug delivery
	NCT03740256	HER2 Positive Solid tumors	1	–	HER2-specific CAR- and Virus-specific T cell therapy	T cell-mediated lysis of virally-infected tumor cells
	NCT04053283	metastatic or advanced epithelial tumours	1	CXCL9/10, IFNα	–	Immune activation and recruitment
	NCT05717712	Diffuse intrinsic pontine glioma	1	IL-12 (cytosolic)	–	Immune activation in the TME
	NCT04217473	Metastatic melanoma	1	TNFα, ιL-2	Adoptive TIL therapy	Immune activation, lymphocyte expansion
Herpes Simplex Virus	NCT05266612	Advanced solid tumors	1		Nivolumab	Immune activation
	NCT04735978	Solid tumors	1	α-CTLA-4, CD40L, 4-1BBL	Nivolumab	Immune activation and evasion of immune suppression
	NCT05223816	Hepatocellular carcinoma	1	IL-12, IL-15, α-PDL1	–	
	NCT05361954	relapsed and refractory solid tumors	1	α-PD1, τGFβR2 decoy, IL-12	–	
Vesicular Stomatitis Virus	NCT03647163	refractory NSCLC or Neuroendocrine Carcinoma	2	IFNβ	Pembrolizumab	Toxicity minimization, immune activation
	NCT03120624	Endometrial cancer	1		Ruxolitnib	

### 5.3 Implications of OV interactions in the solid tumor and therapeutic timing for OV and NK cell combination therapy efficacy

Although arming OVs with immunomodulatory agents that could enhance NK cell intra-tumoral infiltration and activation is promising, the anti-tumor efficacy of oncolytic virotherapy is also impacted by the TME. For example, several studies have shown that OVs can have anti-angiogenic effects by directly infecting tumor vascular endothelial cells leading to vascular shutdown (168). While this phenomenon was previously regarded as beneficial for virotherapy due to sequestering of the OV in the tumor, complete collapse of the vasculature can have a negative effect on immune cell infiltration and drug delivery into the solid tumor (168). OVs such as VSV, VV, and oHSV-1 have been shown to directly infect and replicate in endothelial cells, leading to decreased blood perfusion to the tumor core and increased hypoxic conditions (169–172). This has been mainly attributed to high levels of VEGF in the TME which can sensitize OV replication in vascular endothelial cells (168). To overcome the vascular shutdown induced by OVs, vascular normalization strategies prior to OV infection can be administered. For example, Matuszewska et al. demonstrated that administration of a protein containing the three homologous thrombospondin type 1 repeat domains (3TSR) prior to NDV infection rescued vascular shutdown caused by NDV in ovarian cancer tumors (173). 3TSR binds to the CD36 receptor on endothelial cells preventing endothelial cell proliferation and reduces VEGF production, leading to vascular normalization and enhancing tumor perfusion (173, 174). Indeed, combination therapy with 3TSR and NDV significantly enhanced infiltration of activated NK cells and T cells to the tumor core and led to tumor regression in an advanced-stage ovarian cancer model (173). This work provides further evidence that preventing complete vascular shutdown may be crucial to ensure high immune mediated anti-tumor efficacy and that OVs which do not infect vascular endothelial cells would be preferred candidates for combination with adoptive cell therapies.

OVs can be delivered via systemic or intra-tumoral routes, with systemic administration being preferred when treating hard to reach solid tumors or after cancer metastasis. However, the dense tumor stroma can also be a barrier to tumor cell infection by OVs which could limit OV efficacy during systemic administration. To overcome this, OVs have been engineered to directly target the tumor stroma. Jing et al. demonstrated that using a modified version of MV that can infect and replicate in both stromal and tumor cells resulted in 69% reduction in tumor volume and improved survival in a colon xenograft tumor model (175). Adenoviruses have also been engineered to express proteins like relaxin and decorin which downregulate collagen expression in tumor tissue, enhancing viral spread in the tumor and improving anti-tumor effects (176–178). Co-administration of bacterial collagenase with HSV-1 improved virus distribution and tumor infection in a melanoma model by disrupting fibrillar collagen (179). Other methods to enhance oncolytic viral spread within the tumor core is by targeting collagen through expression of matrix metalloproteinases such as MMP-9 (180). In addition, a phase I clinical trial using an oncolytic adenovirus expressing human hyaluronidase to target hyaluronic acid, facilitating ECM breakdown, showed this strategy reduced tumor stiffness in patients with pancreatic cancer (NCT02045589) (181). Further, OVs can also package proteins that modulate effector cell cytotoxicity against stromal cells. Bi-specific T cell engagers (biTEs) have been designed to specifically direct cytotoxic T cells towards a tumor target. Both, adenoviruses and VV have been used to express biTEs binding to CD3 and mouse or human FAP proteins (182, 183). Locally targeting T cells to FAP+ stromal cells led to high infiltration of T cells to the tumor sites in mouse melanoma and human lung and breast cancer models (182, 183). Further, combination of the biTE secreting adenovirus and intravenous injection of pre-activated T cells led to significant enhancement in survival compared to the monotherapies (182). Using OVs to express and release biTEs in the local tumor environment can be a successful way to overcome potential off-target safety concerns as seen in anti-FAP CAR-T cell strategies. Given the recent development of bi- and tri-functional NK cell engagers (NKCEs) which bind to NK cell activation receptors, there is potential to engineer OVs with NKCEs targeting stromal components similarly to the biTEs discussed (184). Overall, these strategies may not only enhance OV distribution in the tumor enabling systemic administration when tumors are not physically accessible but could also increase intra-tumoral infiltration of adoptively transferred NK cells.

The timing of NK cell administration is an important consideration during combination therapy as NK cells could impede oncolytic virotherapy through elimination of virally infected cells, limiting viral replication and spread. Mathematical modelling was performed to determine the highest efficacy between oHSV-1 and the proteasome inhibitor, Bortezomib, in combination with NK cell therapy (185). The modelling determined that the highest anti-tumor efficacy would be achieved when endogenous NK cells are depleted or when higher numbers of exogenous NK cells are added (185). In addition, they found that higher anti-tumor efficacy against intracranial glioma tumors was achieved when there was a delay between OV infection and exogenous NK cell administration (185). Performing simulations such as these could be useful to inform the rational development of engineered OVs and NK cell combination therapies to maximize the ability of OVs to promote infiltration of NK cells into the tumor.

### 5.4 Other tools for the delivery of immune recruiting and activation agents to the TME

While OVs are one method to induce chemokine production in the tumor, new avenues have emerged which can penetrate the tumor core more efficiently. One strategy involves the use of nanomedicines which are therapeutic carriers that are nanometers in size, typically lower than 100 nm (186, 187). An example of these are lipid nanoparticles (LNPs) or extracellular vesicles such as exosomes which can be loaded to carry similar cargo as OVs to induce the production of chemokines and cytokines from tumors to mediate immune cell recruitment (186, 188). These nanomedicines were originally formulated as a mechanism of delivering chemotherapeutic agents directly to tumors, to prevent off-tumor toxicity (189, 190). However, the widespread use of mRNA based COVID-19 vaccines delivered via nanoparticles has led to an increased interest in investigating how LNPs can be exploited to promote anti-tumor immunity. LNPs are typically manufactured using a profile of lipids, cholesterol, and stabilizing agents such as polyethylene glycol (PEG). After complexing with genetic material, such as mRNA, miRNA, or siRNA, they can be systemically administered to promote therapeutic gene manipulation. These nanomedicines are endocytosed by cells, after which they release their genetic cargo into cells following endosomal maturation allowing efficient delivery of therapeutic cargo (187, 191).

As they often lack tissue specificity, there has been a focus on investigating methods for targeted delivery of these nanoparticles to different tissue sites, including tumors. While direct, intratumoral delivery of such nanomedicines is possible, research has been focused on advancing ease of tumor permeability of systemically delivered nanoparticles to enhance their efficacy (187, 192). Such approaches usually involve conjugating nanoparticles with antibodies against ligands expressed on tumors, to increase tumor targeting and decrease competition for uptake by other organs. Other strategies leverage high expression of certain proteins within the tumor (193–195). For example, nanoparticles can be designed to become active in response to MMP-2, a metalloproteinase highly expressed within the tumor bed (196, 197). Other than passive transport into the tumor bed through blood vessels, nanoparticles can also be actively transported through tumor endothelial cells (198). Further, entry into the tumor by nanoparticles can also be inhibited by the basement membrane of the solid tumor. However, unlike immune cells, nanoparticles that are larger in size (>30 nm) have enhanced entry into the tumor through their uptake by TAMs, which are abundant in the TME and can migrate within the tumor core (199–203). Recent research shows that nanoparticles can exit the tumor through the tumor-draining lymph nodes and re-enter the circulation allowing for re-entry into the tumor (204). This discovery will allow for further modification of nanoparticles to enhance tumor accumulation.

Various candidate therapeutics have been explored for the delivery of different cargoes including cytokines, chemokines, mAbs, and other forms of engineered antibodies such as nanobodies, and other immune-stimulatory molecules such as stimulator of interferon genes (STING) agonists (205–209). STING agonists have emerged as a strategy to activate the cyclic GMP-AMP synthase (cGAS) STING pathway to enhance CD8+ T cell and NK cell infiltration and activation in the TME. The pathway is triggered by sensing of cytosolic double stranded DNA (dsDNA) by cGAS which catalyzes the production of the secondary messenger, cyclic 2’3’-GMP-AMP (cGAMP) (210). cGAMP is the endogenous cyclic dinucleotide (CDN) ligand for STING (210). Pharmacologic activation of the STING pathway has mainly been focused on intra-tumoral administration of STING ligands such as natural CDNs like cGAMP, synthetic CDNs, and non-CDN molecules.

STING agonists induce potent local and systemic anti-tumor immunity through STING activation in tumor cells, CAFs and tumor infiltrating DCs and macrophages (211, 212). STING activation in these cells leads to the production of type 1 IFNs and CXCL9 and CXCL10 chemokines, enhancing the migration of CXCR3-expressing CD8+ T cells and NK cells (209, 213–216). Additionally, type 1 IFNs can promote cross-presentation of antigens to CD8+ T cells and induce expression of IL-15 and the IL-15 receptor α (IL-15Rα) by DCs leading to NK cell activation (217). Given the upregulation of CXCR3 on *ex vivo* expanded NK cells and their ability to migrate towards tumors expressing exogenous CXCL10, STING agonists could enhance their tumor homing (77). STING therapy also can normalize the aberrant tumor vasculature through activation of STING on endothelial cells, leading to reduced tumor vessel density and improving pericyte coverage thereby increasing intratumoral CD8+ T cell infiltration (218). STING agonist therapy is currently being evaluated preclinically as a combination therapy with adoptive cell therapies such as CAR-T cell and NK cells. Xu et al. demonstrated that subcutaneous delivery of STING agonists enhanced the infiltration and persistence of CAR-T cells in an orthotopic mouse breast tumor model through increased expression of CXCL9 and CXCL10 by myeloid cells in the TME (219). However, STING agonists can be toxic to human T cells which will limit their combination (220). Interestingly, the same effect is not observed with human naïve or expanded NK cells which may be partly due to rapid degradation of STING after STING agonist stimulation (221). Da et al. showed that cGAMP can directly activate the human NK-92 cell line and increase their anti-tumor function against pancreatic tumors and enhanced migration of CCR5-expressing NK-92 cells *in vitro (* 222). Knelson et al. showed that STING agonist therapy can also enhance migration and cytotoxicity of primary NK cells in a more physiological relevant model using patient-derived organotypic tumor spheroids (221). The enhanced tumor targeting and migration which was dependent on release of CXCR3 ligands by STING agonist activated tumor cells. STING agonists can also be combined with cytokine therapy to improve overall NK cell responses. Administration of a high affinity IL-2 cytokine after intra-tumoral CDN injection induced higher expression of cytotoxic effector molecules by tumor infiltrating NK cells in HLA class 1 deficient tumors and enhanced infiltration of NK cells to the distal tumor (223).

Using nanocarriers as a delivery method for STING-agonists is under active investigation to enhance their half-life by preventing their degradation by proteases and decreasing non-specific uptake which can cause apoptosis of STING agonist sensitive cells like T cells and B cells (224–226). In addition, delivery using nanocarriers improves target cell uptake since CDNs are hydrophilic and negatively charged they do not passively enter the cellular membrane and are instead transported using important channels (227, 228). For example, systemic delivery of CDNs using PEGylated lipid nanodiscs (LNDs) has shown improved CDN delivery in the tumor and uptake by DCs in tumor draining lymph nodes enhancing STING-agonist mediated anti-tumor immunity (229). Although STING agonist delivery using OVs has been recently reported, enhanced tumor entry to the tumor core by nanocarriers is an advantage (230). Overall, STING agonists have the potential to improve the efficacy of NK cell-based therapies by promoting their infiltration into the tumor and further activating the anti-tumor responses without affecting their viability.

A few studies have previously tried to leverage the use of engineered nanotherapeutics to promote tumor immune cell infiltration. One study had described the delivery of a CCL2-binding single-domain antibody to the TME of TNBC tumors in mice (231). CCL2 is of interest as tumor-associated adipocytes, a major component of the tumor stroma in TNBC, can secrete CCL2 to promote infiltration of other pro-tumoral cells, such as MDSCs, into the TME (232). The CCL2 antibody was encoded in a plasmid encapsulated in a lipid-protamine-DNA nanoparticle and was delivered intravenously. This led to a higher anti-tumor efficacy than an anti-CCL2 monoclonal antibody alone and was able to significantly inhibit TNBC tumor progression. CCL2 blockade also led to an increase in CD3+ lymphocyte recruitment by promoting an increase in production of CXCL9 and CXCL10, as well as other inflammatory cytokines like IL-12 and IFN-γ (232). Importantly, this strategy was relatively tumor-specific and did not lead to tissue damage to other organs.

An alternative approach is the expression of surface ligands on nanomedicines to improve the efficacy of immunotherapies. For example, some studies leverage the expression of surface ligands on nanoparticles to trigger expansion and activation of cells *in vivo*. One study has shown that complexing an IL-15 super-agonist complex selectively expands T cells in tumors and enabled higher administration of IL-15 to boost CAR-T cell efficacy in mice (233). Membrane-bound cytokines, such as IL-15 and IL-21, in combination with adoptive immunotherapy could improve the activation and persistence of NK cell immunotherapies. Further, coating nanoparticles with other molecules such as membrane-bound chemokines and cytokines or ligands that block immune-checkpoint ligands has been investigated. One study conjugated anti-PD-L1 antibodies to promote T cell expansion within the tumor. As ICB therapy is often limited by the penetration of these antibodies into the tumor, this strategy acts to effectively trigger anti-PD-L1 engagement at a deeper level inside the tumor bed (234–236). Another study showed nanoparticle solid tumor penetration when using an anti-CCR7-loaded nanoparticle to sequester CCR7. CCR7 is known to be involved in mediating TNBC metastasis to the lymph nodes (237, 238). This strategy prevented metastasis in a murine syngeneic melanoma model (237, 238). While an anti-CCR7 antibody elicited immunosuppression by counteracting immune cell migration, CCR7-loaded nanoparticles are not limited in the same way (237, 238). CCR7-loaded nanoparticles would limit their effects directly to the tumor, decreasing bystander effects of the therapy compared an anti-CCR7 antibody which works systemically. Overall, when considering strategies to induce immune cell infiltration into the tumor bed, nanoparticles hold promise due to their ability to enter the tumor bed and ability to be targeted to tumors.

## 6 Concluding remarks

**Figure 3:**
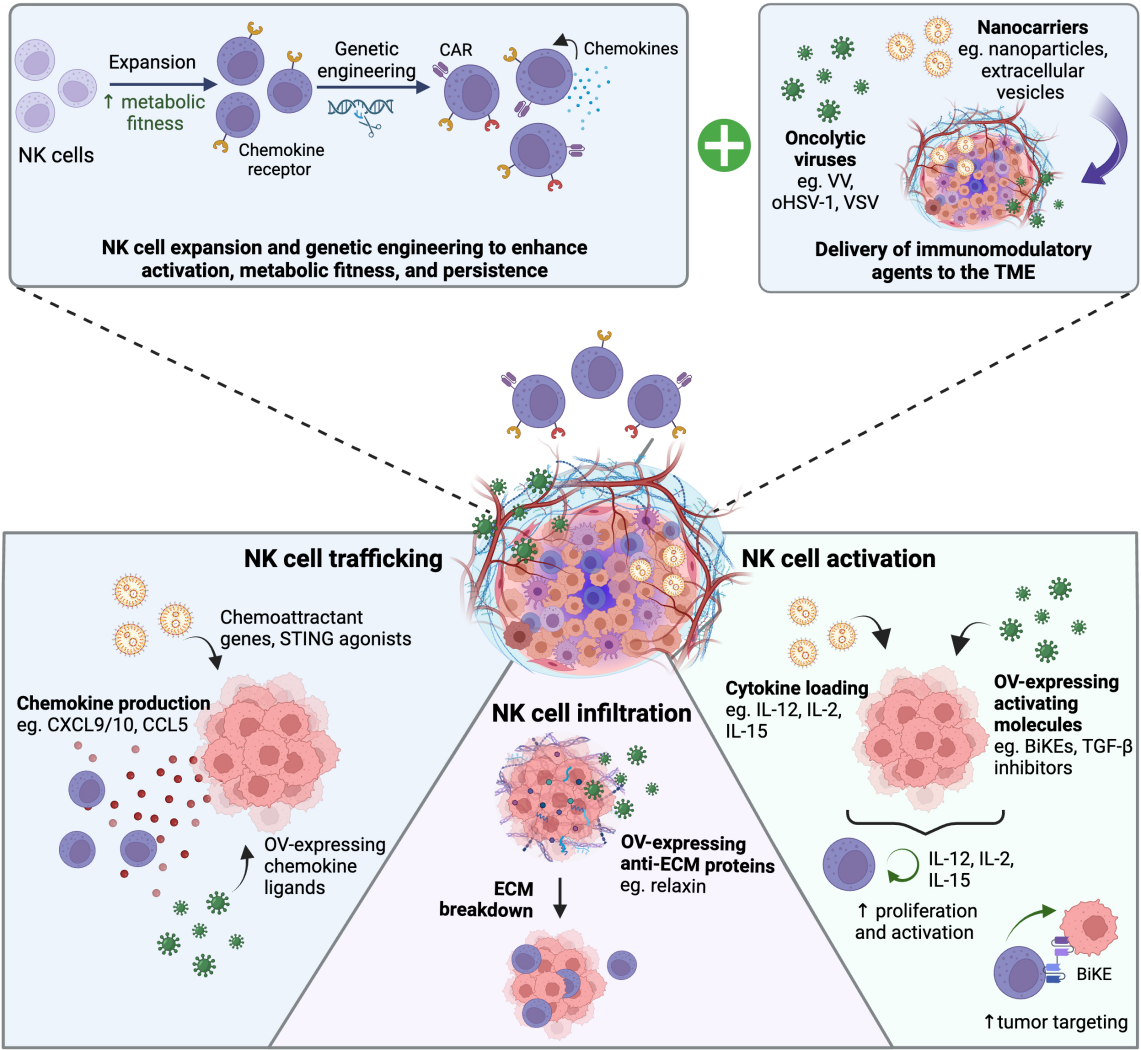
Delivery of immunomodulatory agents to the TME using oncolytic viruses or nanocarriers to pull and boost NK cell-based adoptive cell therapies. Schematic showing how engineered oncolytic viruses (OVs) or nanocarriers can synergize with NK cell-based adoptive cell therapies to enhance NK cell trafficking, infiltration, and activation in the solid tumor. Highlighted are different immunomodulatory agents that can be delivered in the tumor to induce chemokine production in the tumor, break down the extracellular matrix (ECM), or promote NK cell anti-tumor responses. Created with BioRender.com.

NK cell-based cancer immunotherapies have emerged as a promising cancer treatment option due to NK cell’s natural ability to recognize and kill cancer cells. *Ex vivo* activated and expanded NK cells have shown high clinical efficacies against hematologic malignancies. Further, advancements in NK cell genetic engineering methods have enabled the efficient generation of highly potent and safe CAR-NK cells which have shown promising safety and efficacy for treating CD19-expressing cancers. However, extending NK cell immunotherapies against solid tumors has been met with various challenges including impaired NK cell infiltration into solid tumor sites and NK cell dysfunction in the TME. While NK cell expansion using K562-mb-IL-21 feeder cells and IL-15 stimulation can enhance NK cell metabolic fitness allowing them to thrive in the TME, efficient homing of adoptively transferred NK cells to the tumor remains to be addressed. The tumor promotes the recruitment of structural and immune cells, which are reprogrammed into a suppressive phenotype within the tumor bed and support tumor growth. Physical barriers posed by the tumor also prevent recruitment of immune effector cells and prevent their permeation into the tumor core to elicit their activity. Various strategies have been developed to target the dense tumor stroma and aberrant tumor vasculature for improved drug delivery and immune cell infiltration into solid tumors. However, few studies directly explore the impact that these strategies have on NK cells, and instead focus generally on lymphocyte recruitment. In addition, the therapeutic efficacy of such strategies relies on stimulation of immune cells other than NK cells, particularly tumor specific T cells, and there are still very limited studies combining these strategies with NK cell adoptive cell therapies. As shown in **Figure 3**, reprogramming the TME with immunomodulatory agents to promote adoptive cell therapy recruitment is a promising strategy to enhance NK cell infiltration and anti-tumor activity. The ability of OVs to selectively express high concentrations of immunomodulatory agents within the tumor make them an attractive platform for developing novel engineered OVs to enhance intra-tumoral infiltration of adoptively transferred NK cells. An advantage of OVs is that expression of their therapeutic payload is restricted to their replication within the TME, which can limit toxicities seen with systemic administration of immunomodulatory agents. This strategy has been shown to promote enhanced recruitment and prolonged tumor clearance when combined with engineered NK cell therapies, through OV expression of chemokines and activating cytokines such as IL-15. Further, OVs without the capacity to induce complete vascular shutdown that are engineered to express stroma normalization agents and NK cell-specific chemokines could drastically enhance access of adoptively transferred NK cell to the tumor bed. However, careful considerations must be taken for rational design of such combination therapies given the complex interplay between OVs and NK cells. For example, optimizing the timing of OV and NK cell administration will be important due to NK cell’s ability to destroy infected tumor cells and prevent viral spread. Further, many translational barriers remain for production of clinically applicable OVs with regards to viral delivery, spread, and antiviral immunity. Recent advancements have shown that it is possible to engineer OVs such as VV to regulate their replication and control the timing and dose of therapeutic payload using inducible expression systems to enhance safety of OVs (239). Additionally, immunomodulatory proteins can also be introduced into the tumor core using nanomedicines such as NPs and may be safer than OV administration. While antiviral immunity would limit the repeated use of OVs, NPs can be used for the delivery of multiple proteins at different points during a therapeutic regimen. However, non-specific uptake of NPs by many different organs including the reticuloendothelial system in the liver can limit targeted tumor delivery. While this can be overcome through engineering NPs with tumor specific antibodies or proteins, efficient tumor delivery can also be hampered by the ECM. The ability to engineer both NK cells and tumor-targeting modalities such as OVs and NPs enables unlimited modifications to fully harness the anti-tumor functions of NK cell therapies against solid tumors with limited treatment options.

## Author contributions

AP: Conceptualization, Visualization, Writing – original draft, Writing – review & editing. JM: Writing – original draft, Writing – review & editing. ER: Writing – original draft. TR: Writing – review & editing. AG: Supervision, Writing – review & editing. AA: Funding acquisition, Supervision, Visualization, Writing – review & editing.
